# *Candida albicans*: The Ability to Invade Epithelial Cells and Survive under Oxidative Stress Is Unlinked to Hyphal Length

**DOI:** 10.3389/fmicb.2017.01235

**Published:** 2017-07-17

**Authors:** Paloma K. Maza, Alexis Bonfim-Melo, Ana C. B. Padovan, Renato A. Mortara, Cristina M. Orikaza, Lilian M. Damas Ramos, Tauany R. Moura, Frederico M. Soriani, Ricardo S. Almeida, Erika Suzuki, Diana Bahia

**Affiliations:** ^1^Disciplina de Parasitologia, Departamento de Microbiologia, Imunologia e Parasitologia, Escola Paulista de Medicina, Universidade Federal de São Paulo São Paulo, Brazil; ^2^Laboratório Especial de Micologia, Disciplina de Infectologia, Departamento de Medicina, Escola Paulista de Medicina, Universidade Federal de São Paulo São Paulo, Brazil; ^3^Departamento de Microbiologia e Imunologia, Universidade Federal de Alfenas Alfenas, Brazil; ^4^Laboratório de Micologia Médica e Microbiologia Bucal, Departamento de Microbiologia, Universidade Estadual de Londrina Londrina, Brazil; ^5^Departamento de Biologia Geral, Instituto de Ciências Biológicas, Universidade Federal de Minas Gerais Belo Horizonte, Brazil

**Keywords:** *Candida albicans*, hyphae, HeLa cells, actin, cortactin, cell signaling

## Abstract

In its hyphal form, *Candida albicans* invades epithelial and endothelial cells by two distinct mechanisms: active penetration and induced endocytosis. The latter is dependent on a reorganization of the host cytoskeleton (actin/cortactin recruitment), whilst active penetration does not rely on the host's cellular machinery. The first obstacle for the fungus to reach deep tissues is the epithelial barrier and this interaction is crucial for commensal growth, fungal pathogenicity and host defense. This study aimed to characterize *in vitro* epithelial HeLa cell invasion by four different isolates of *C. albicans* with distinct clinical backgrounds, including a *C. albicans* SC5314 reference strain. All isolates invaded HeLa cells, recruited actin and cortactin, and induced the phosphorylation of both Src-family kinases (SFK) and cortactin. Curiously, L3881 isolated from blood culture of a patient exhibited the highest resistance to oxidative stress, although this isolate showed reduced hyphal length and displayed the lowest cell damage and invasion rates. Collectively, these data suggest that the ability of *C. albicans* to invade HeLa cells, and to reach and adapt to the host's blood, including resistance to oxidative stress, may be independent of hyphal length.

## Introduction

*Candida albicans* is a polymorphic opportunistic human pathogen that takes on the most common morphologies of yeast, pseudohyphae, and true hyphae. All morphology types are found during infection; however, hyphae are generally the invasive form of *C. albicans* (Jacobsen et al., 2012). *C. albicans* frequently causes superficial infections in mildly immunocompromised patients, but in severely immunocompromised or critically ill patients, this fungus may cause life-threatening, disseminated disease. The *Candida* genus is associated with approximately 80% of nosocomial fungal infections, representing the major cause of fungemia showing a high mortality rate (40–60%) (Perlroth et al., 2007; Doi et al., 2016).

For most microbial pathogens, adherence to epithelial surfaces is the first step to initiating an infection, and is even a prerequisite for their survival and eventual distribution. After attachment, many pathogens invade host cells (Filler and Sheppard, 2006). With regard to *C. albicans*, this fungus uses various mechanisms of pathogenicity: escape from host immune responses, morphological change (yeast–to–hypha transition) and invasion, which is supported by hyphae-associated factors such as adhesion molecules and the secretion of hydrolytic enzymes (Wilson et al., 2009).

In order to infect mucosal tissues, gain access to blood and subsequently escape from the bloodstream, *C. albicans* must interact with epithelial and endothelial cells (Davis, 2009). The invasion of non-professional phagocytic host cells by *C. albicans* can occur through two mechanisms: induced endocytosis and active penetration. The latter mechanism does not rely on the host's cellular machinery, but possibly on physical pressure applied by the advancing hyphal tip and the secretion of extracellular hydrolases (Wilson et al., 2009).

In contrast, invasion via induced endocytosis is dependent on host cell actin microfilament dynamics. *C. albicans* stimulates epithelial cells to produce pseudopodia-like structures that surround the fungus and cause its uptake. In endocytosis, induced proteases of *C. albicans* have little or no role (Frank and Hostetter, 2007). This highly effective invasive mechanism is predominantly triggered by the interaction between a fungal adhesin (Als3) (Phan et al., 2007) and either human E-cadherin or the epidermal growth factor receptor 2 (Her2) on epithelial cells (Zhu et al., 2012). Such interactions stimulate actin rearrangement in host cells and the subsequent internalization of fungal cells (Phan et al., 2007; Zhu et al., 2012; Yang et al., 2014). In addition, *C. albicans* exploits clathrin-dependent endocytosis, which requires dynamin and cortactin, to invade human epithelial cells (Moreno-Ruiz et al., 2009). Cortactin is involved in actin-related cellular processes ranging from lamellipodium protrusion and extracellular matrix degradation to the uptake of intracellular pathogens such as bacteria and parasites (Chen et al., 2003; Bonfim-Melo et al., 2015). Cortactin interacts with the Arp2/3 complex, newly formed actin filaments and a variety of actin-binding/regulation proteins (Daly, 2004); it is also regulated by phosphorylation (Martinez-Quiles et al., 2004). Oral and vaginal epithelial cells recognize both the yeast and hyphal forms of *C. albicans*. As a consequence, they activate cellular signaling mechanisms, including mitogen-activated protein kinase (MAPK), nuclear factor kappa-light-chain-enhancer of activated B cells (NF-κB), and phosphatidylinositol 3-kinase (PI3K) pathways (Moyes et al., 2010, 2014, 2015; Naglik et al., 2014).

With regard to induced endocytosis, our group has previously shown that extracellular amastigote (EAs) infective forms of *Trypanosoma cruzi* seem to share with *C. albicans* a similar pathway for entry into the host cell. Both events occur via an actin-dependent mechanism and involve cortactin (Moreno-Ruiz et al., 2009; Bonfim-Melo et al., 2015). In addition, *T. cruzi* recruited actin and cortactin to sites of adherence and invasion into HeLa cells and also selectively induced cortactin phosphorylation during cell invasion (Bonfim-Melo et al., 2015). However, possible cortactin phosphorylation in response to *C. albicans* invasion into non-phagocytic cells, such as HeLa cells, has never been assessed. We propose that epithelial cells represent the first obstacle for *Candida* invasion which probably will determinate the fate of this infection. We would like to consider invasion in a broad spectrum which includes crossing the endothelial barrier, reaching and adapting to blood.

In this study, we verified the ability of different *Candida albicans* isolates (the reference strain SC5314 and three *C. albicans* clinical isolates) to invade HeLa epithelial cells. First, we analyzed hyphal growth, cell internalization and damage rates and resistance to oxidative stress. Furthermore, we investigated HeLa cell cortactin/actin recruitment and Src-family kinases (SFK)/cortactin phosphorylation/activation in response to these isolates. Our data showed that all isolates are capable of invading HeLa cells, recruiting actin/cortactin and inducing SFK and cortactin phosphorylation. Interestingly, L3881 isolate, that has a natural *HWP1* mutation, the shortest hyphae and the lowest internalization and damage rates, was able to reach the blood of a patient and to survive under nutritional and oxidative stress, indicating that the latter phenomenon was independent of hyphal length or damage/invasion rate.

## Materials and methods

### *Candida* strains

*C. albicans* isolates were obtained from various sources: 997,5 g was isolated from the blood of a deceased patient (Chaves et al., 2012), L3881 (LEMI #L3881, Padovan et al., 2009) was isolated from blood of a patient who had candidemia which was cured clinically, and L3837 was an isolate from the oral cavity (oropharynx, LEMI# L3837, non-hematogenic). *C. albicans* SC5314 is a reference strain. The isolates were kindly provided by LEMI (Laboratorio Especial de Micologia, Universidade Federal de São Paulo, Brazil). *C. albicans* isolates were grown at 30°C on YPD (1% [w/v] yeast extract; 2% [w/v] peptone, 2% [w/v] dextrose) medium plates. Before performing all experiments, a single colony of *C. albicans* isolates was inoculated in YPD liquid media and incubated in an orbital shaker at 30°C for 24 h. Then, *C. albicans* cells were washed with phosphate buffered saline, pH 7.2 (PBS), harvested by centrifugation, and counted in a hemocytometer chamber.

### Cell culture

HeLa cells (human epithelial cells from a fatal cervical adenocarcinoma) were obtained from the Instituto Adolfo Lutz (São Paulo, SP, Brazil) and maintained in complete RPMI medium with 10% fetal bovine serum (FBS) supplemented with antibiotics (10 μg/mL streptomycin and 100 U/mL penicillin; Sigma–Aldrich, St. Louis, MO, USA) in a humidified atmosphere at 37°C and 5% CO_2_.

### Antibodies

Rabbit anti-pT202/Y204 ERK1/2 (#9101S) and rabbit anti-pY416 SFK (#2101S) were obtained from Cell Signaling, Beverly, MA, USA. Rabbit anti-pY466 cortactin (PA114115) was obtained from Thermo Scientific, Waltham, MA, USA. Mouse monoclonal anti-β-actin (AC-74, A2228) was obtained from Sigma-Aldrich. Mouse monoclonal anti-cortactin (A-4; sc-55578) was from Santa Cruz Biotechnology (Santa Cruz County, CA, USA). The secondary antibody, goat anti-mouse AlexaFluor® 488 (A11001), was from Invitrogen, Carlsbad, CA, USA. Goat anti-rabbit IgG peroxidase (A6154) and goat anti-mouse IgG peroxidase (A4416) were obtained from Sigma–Aldrich.

### Hyphal growth assay

*C. albicans* blastospores (3 × 10^5^) were seeded in complete RPMI medium at 37°C and 5% CO_2_ for 2 h on glass coverslips in a 24-well plate. *C. albicans* cells were fixed with 4% paraformaldehyde, washed with phosphate buffered saline, pH 7.2 (PBS), and analyzed under a BX51 microscope (Olympus, Tokyo, Japan) with a 60× objective. Random images from 10 fields were acquired by a DP71 camera (Olympus) and hyphal lengths were measured using ImageProPlus 6.2 software (Media Cybernetics, Rockville, MD, USA). Data were treated and plotted using Office Excel and Prism 5 (GraphPad, San Diego, CA, USA). Experiments were performed in triplicate.

### Cell internalization assay

To evaluate cell internalization by *C. albicans* strains, HeLa cells were cultured in complete RPMI medium on glass coverslips treated with acetic acid until they form confluent monolayers (80–100% confluence). After washing with PBS, monolayers were seeded with 2 × 10^5^ *C. albicans* blastospores in RPMI 1640 medium without FBS for 2 h at 37°C in 5% CO_2_. Coverslips were fixed with 4% paraformaldehyde for 30 min. To stain *C. albicans* cells localized only to the outside of epithelial cells, coverslips were incubated with 25 μg/mL Concanavalin A–fluorescein conjugate (Invitrogen) in PBS for 45 min. After permeabilization with 0.1% Triton X-100 for 15 min, fungal cells localized outside and inside epithelial cells were stained with 10 μg/mL Calcofluor White (Sigma-Aldrich) in 0.1 M Tris hydrochloride (pH 9.0) for 20 min. After each step, HeLa cells were washed three times with PBS. Coverslips were mounted on slides with 50% glycerol and analyzed under a fluorescence microscope (Zeiss, Jena, Germany; Axio Observer Research microscope). This method allowed differentiation between hyphae of the fungus that were located in the external medium and hyphae that invaded the host cell (intracellular) (Almeida et al., 2008). Three experiments in duplicate were analyzed and 10 randomly chosen fields per coverslip were evaluated.

### Epithelial cell damage assay

To evaluate the cell damage caused by *C. albicans* strains, HeLa cells were incubated in complete RPMI medium on glass coverslips treated with acetic acid until they form confluent monolayers (80–100% confluence). Afterwards, 1 mL PBS was added and removed from each well to wash the cells. Finally, the monolayers were co-incubated with 10^6^ *C. albicans* blastospores in 1 mL RPMI 1640 medium without FBS for 8 h at 37°C and 5% CO_2_. To quantify cell damage, supernatants were removed and the amount of lactate dehydrogenase (LDH) released into the medium was determined using LDH Cytotoxicity Assay Kit (Cayman Chemical, Ann Arbor, MI, USA) following manufacturer's instructions. Samples containing only epithelial cells or fungal cells alone were used as controls.

### Phenotypic characterization

*C. albicans* blastospores of the SC5314 reference strain and L3881 (10^2^–10^6^) were seeded in YPD medium plates containing different stressor agents: 200 mM CaCl_2_; 2 mM FeSO_4_; 200 mM MgCl_2_; 200 mM NaCl; 3 mM ethylenediaminetetraacetic acid (EDTA); 0.125 μg/mL 4-nitroquinoline N-oxide (4NQO); 50 μM camptothecin (CPT); 0.005% methyl methanesulfonate (MMS); 5 mM hydrogen peroxide (H_2_O_2_); and 1.25 mM paraquat (Pqt). Plates were incubated for 24–48 h at 30°C.

### Host molecules recruitment, immunofluorescence, and confocal microscopy

HeLa cells were cultured in complete RPMI medium on glass coverslips treated with acetic acid until they form confluent monolayers (80–100% confluence). After washing with PBS, HeLa cells were seeded with 10^5^ *C. albicans* blastospores in RPMI 1640 medium without FBS for 2 h at 37°C in 5% CO_2_. Coverslips were washed with PBS, fixed with 3.5% paraformaldehyde for 15 min at room temperature and sequentially incubated with Calcofluor White (10 μg/mL), phalloidin-TRITC (10 ng/mL), anti-cortactin (20 μg/mL) and anti-mouse AlexaFluor® 488 (20 μg/mL) secondary antibodies diluted in PGNS solution (PBS pH 7.2 with 0.1% gelatin [Sigma–Aldrich], 0.1% sodium azide [Sigma–Aldrich] and 0.2% saponin [Sigma–Aldrich]) at room temperature in an humidified chamber for 1 h. Coverslips were mounted on slides in pH 9.0 buffered glycerol solution with 9 mM *p-*phenylenediamine. Then, HeLa–*C. albicans* interactions were evaluated and imaged in a TCS SP5 II Tandem Scanner (Leica, Wetzlar, Germany) confocal microscope with a 63 × 1.40 NA objective. Image processing and analysis were performed with Imaris v.7.4.2 software (Bitplane, Belfast, Ireland) or ImageJ (https://imagej.nih.gov/ij/).

### Signaling and western blot assays

Western blots were performed essentially as described by Bonfim-Melo et al. (2015). HeLa cells were seeded with 10^8^ *C. albicans* blastospores and incubated for various time intervals (ranging from 0 to 180 min) at 37°C in 5% CO_2_. Protein extracts were quantified using Micro BCA Protein Assay Kit (Thermo Scientific), according to the manufacturer's instruction.

### Statistical analysis

All results are presented as the mean ± standard deviation (s.d.). Differences were considered statistically significant when *p* < 0.05, as determined by one-way ANOVA and Tukey's multiple comparison post-test. Analyses and graphs were created using Prism 5 (GraphPad, San Diego, CA, USA) software.

## Results

### Hyphal growth, internalization, and damage rates

**Figure 1:**
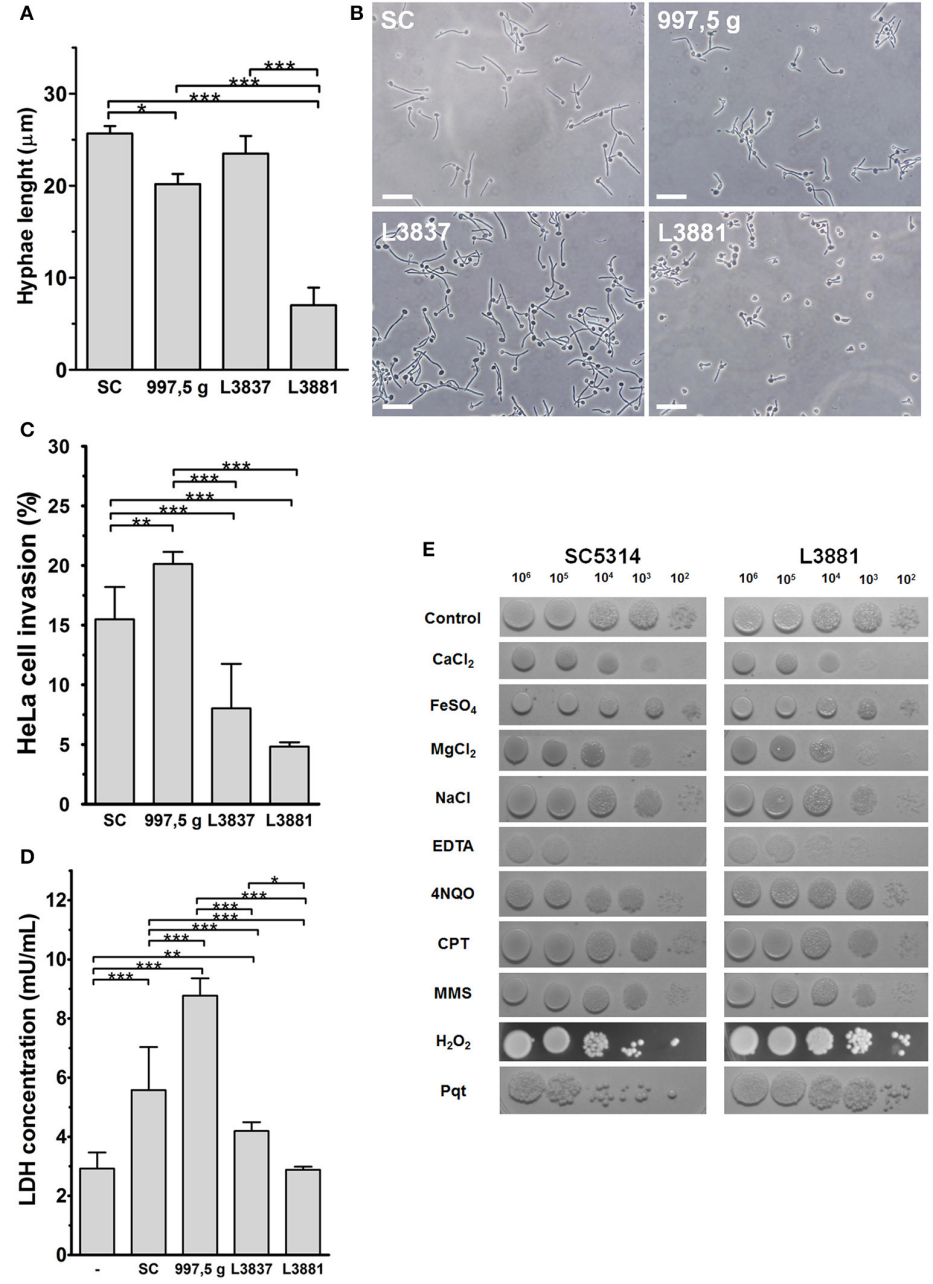
Characterization of HeLa epithelial cell invasion by four *C. albicans* isolates. **(A,B)** The blood isolate L3881 displayed a smaller hyphal length. Blastospores from SC5314, 997,5 g, L3881 and L3837 isolates were seeded on glass coverslips for 2 h at 37°C and 5% CO_2_, washed, fixed and their hyphal length measured under optical microscopy. **(A)** Hyphal length of all isolates used (mean ± s.d., ^*^p < 0.05 and ^***^p < 0.001). **(B)** Representative fields of each isolate observed under optical microscopy, showing the overall aspect and size of hyphal length. Bar = 20 μm. **(C,D)** The 997,5 g and L3881 blood isolates displayed the highest and lowest invasion index and cytotoxicity for HeLa cells, respectively. **(C)** Blastospores from all isolates were seeded with HeLa cells for 2 h at 37°C and 5% CO_2_, washed, fixed, stained for intracellular/extracellular hyphae discrimination and the cell invasion indexes evaluated under epifluorescence microscopy. The 997,5 g and SC5314 isolates displayed higher invasion indexes compared to the other isolates (L3837 and L3881; mean ± s.d. ^**^p < 0.01 and ^***^p < 0.001). **(D)** For cytotoxicity assays, HeLa cells were seeded with blastospores for 8 h at 37°C and 5% CO_2_, washed and the amount of lactate dehydrogenase in culture supernatants was quantified by a commercial kit. L3881 did not induce toxicity in HeLa cells while the 997,5 g strain induced significantly higher cytotoxicity compared to other isolates (mean ± s.d., ^*^p < 0.05, ^**^p < 0.01, and ^***^p < 0.001). **(E)** L3881 showed more plasticity under stress. 10^2^–10^6^ blastospores of the SC5314 reference strain and L3881 blood isolate were seeded on plates containing different stressor agents for 24–48 h at 30°C. Fungi were cultured in the presence of 200 mM CaCl_2_; 2 mM FeSO_4_; 200 mM MgCl_2_; 200 mM NaCl; 3 mM ethylenediaminetetraacetic acid (EDTA); 0.125 μg/mL 4-nitroquinoline N-oxide (4NQO); 50 μM camptothecin (CPT); 0.005% methyl methanesulfonate (MMS); 5 mM hydrogen peroxide (H_2_O_2_); and 1.25 mM paraquat (Pqt). All assays are representative of triplicate experiments.

Hwp1 (hyphal wall protein 1) is a well-characterized *C. albicans* cell surface protein, expressed only on hyphae, which mediates binding to epithelial cells. The isolate L3881 is a *HWP1-2* homozygous strain that showed reduced *HWP1* expression, biofilm production, and hyphae formation (Padovan et al., 2009). In fact, we verified that *C. albicans* L3881 produced the shortest hyphae and in HeLa cells produced the lowest internalization and damage rates when compared to other strains (Figures 1A–C). The L3837 oral strain showed similar hyphal lengths to the SC5314 reference strain (Figures 1A,B). HeLa cell internalization and cellular damage rates were lower for L3837 than for SC5314 (Figures 1C,D). The 997,5 g isolate also displayed a similar hyphal length when compared to the reference isolate, SC5314. However, 997,5 g displayed the highest cell internalization and cell damage rates (Figures 1C,D) in this study. The characteristics of all four *Candida* species are summarized in the Supplementary Table 1 (Supplementary Material).

### The L3881 isolate showed the poorest internalization fitness but with a high resistance to oxidative and nutritional stress

To explore possible physiological alterations, we tested the SC5314 reference strain and the L3881 blood isolate and analyzed their ability to deal with different kinds of stresses (Figure 1E). SC5314 and L3881 strains demonstrated similar sensitivities in the presence of ions such as calcium, magnesium and iron, and during osmotic stress (NaCl). On the other hand, in the presence of ethylenediaminetetraacetic acid (EDTA), a chelator of bivalent ions, L3881 isolate displayed a more resistant phenotype, indicating lower nutritional requirements needed for maintaining growth. In the presence of DNA damage or stress agents of replication (4-nitro-quinoline-N-oxide [4NQO]; camptothecin [CPT] and methylmethanesulfonate [MMS]), both strains showed similar phenotypes in response to functional mechanisms of DNA repair. SC5314 and L3881 isolates were also tested in the presence of oxidative stress inducers such as H_2_O_2_ and Paraquat (Pqt). Paraquat is a potent oxidative stress inducer because it greatly increases reactive oxygen species (ROS) production and inhibits the regeneration of reducing equivalents and compounds necessary for maintaining the redox status. Surprisingly, L3881 the *HWP1-2* homozygous strain with reduced *HWP1* expression with shortest hyphae and the lowest internalization and damage rates in HeLa cells was found to be more resistant to oxidative stress (Figure 1E).

### Different clinical isolates of *C. albicans* invade HeLa cells and recruit actin and cortactin

**Figure 2:**
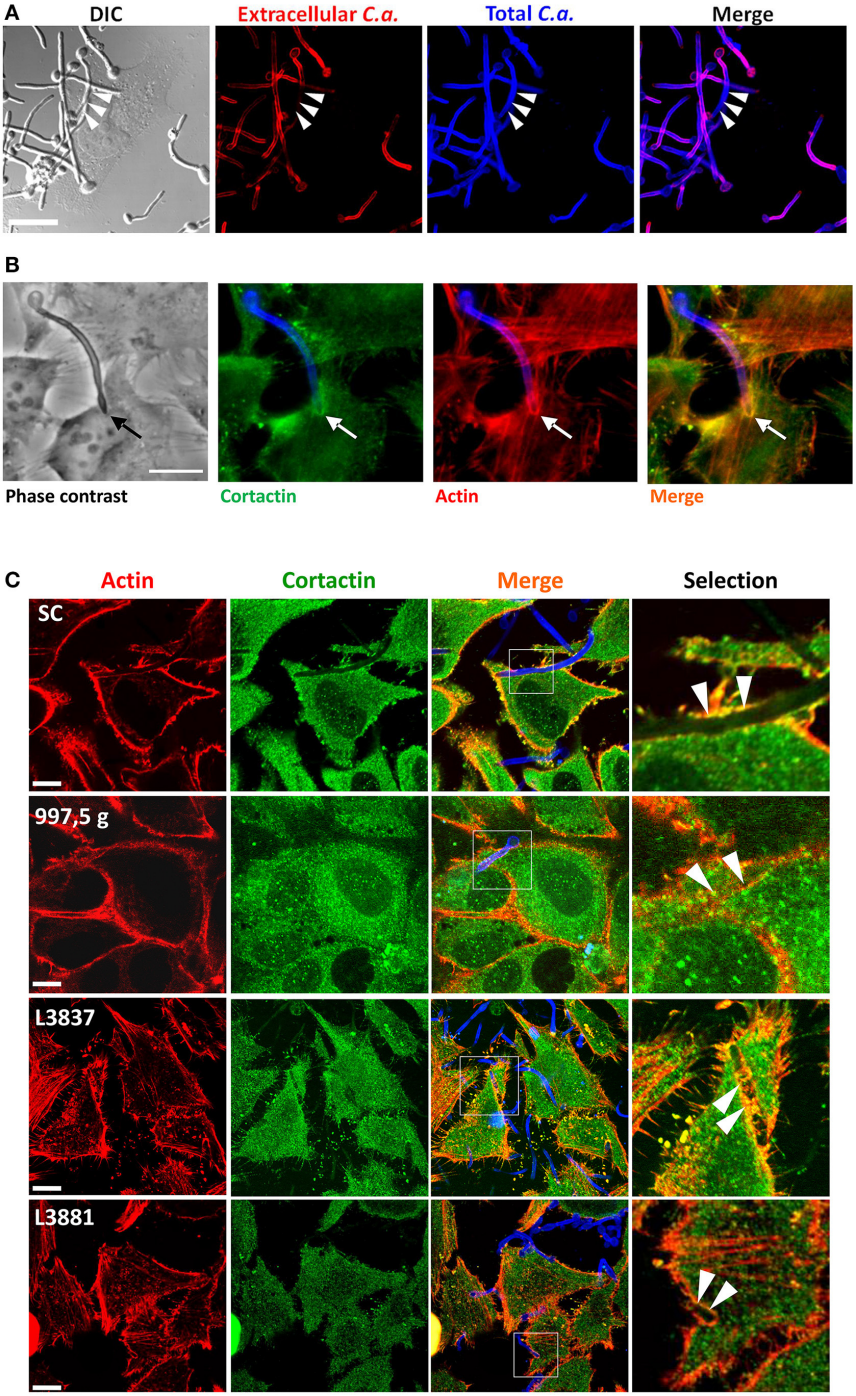
The hyphae of all four *C. albicans* isolates induced the recruitment and colocalization of actin and cortactin. **(A)** *C. albicans* SC5314 strain invaded HeLa cells. *C. albicans* SC5314 blastospores were seeded with HeLa cells for 2 h at 37°C and 5% CO_2_. Extracellular hyphae were stained with Concanavalin A–rhodamine (red), and, after permeabilization, extra- and intracellular hyphae were stained with Calcofluor White (blue). Bar = 10 μm. The arrows show hyphae internalization. **(B)** Interaction of HeLa cells with *C. albicans* SC5314 led to the recruitment and colocalization of actin and cortactin. *C. albicans* SC5314 blastospores were seeded for 2 h with HeLa cells and stained with phalloidin-TRITC (actin: red-orange), Calcofluor white (fungal cell wall: blue) and anti-cortactin antibody followed by Alexa Fluor-488® conjugated secondary antibody (cortactin: green). Epifluorescence microscopy. Bar: 10 μm. The arrows clearly show the recruitment and colocalization of both molecules at the hyphae invasion site in HeLa cells. **(C)** The four *C. albicans* isolates recruit actin and cortactin. Blastospores from SC5314, 997,5 g, L3881 and L3837 isolates were seeded with HeLa cells in RPMI medium for 2 h at 37°C and 5% CO_2_, washed, fixed, immunostained for cortactin, labeled with phalloidin for host actin and evaluated by confocal microscopy. Invading hyphae from all four isolates were able to induce the recruitment and colocalization of actin (red) and cortactin (green) to the proximity of the hyphal invasion sites (arrowheads). Bar = 20 μm.

After observing distinct levels of cell cytotoxicity and invasiveness promoted by different isolates, we then evaluated whether host actin and cortactin could be recruited by these isolates and also be differentially modulated. By staining hyphae, inside and outside HeLa cells, with Calcofluor White and by using Concanavalin A to stain hyphae that were not internalized, we tested the ability of the *C. albicans* SC5314 reference strain to invade HeLa cells. Figure 2A clearly shows the invasion of HeLa cells by *C. albicans* hyphae. Figure 2B shows that cortactin was recruited to host cell ruffles by *C. albicans* SC5314 with a similar distribution to that of F-actin recruitment. Actually, all *C. albicans* isolates recruited cortactin and actin, and both molecules colocalized at sites of hyphae internalization (Figure 2C).

### Biphasic activation of ERK kinase pathway and induction of cortactin-phosphorylated SFK

**Figure 3:**
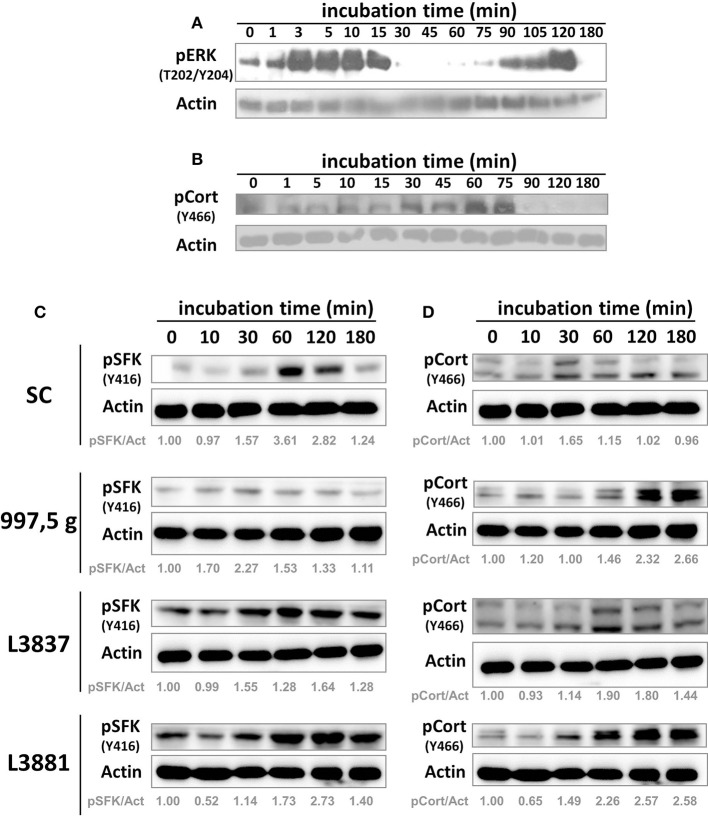
*C. albicans* SC5314 strain induced host cell biphasic ERK 1/2 activation and cortactin phosphorylation by SFKs. HeLa cells were incubated with blastospores from the SC5314 strain for the indicated time points. After incubation, HeLa cells were harvested, lysed, and their protein lysates separated by sodium dodecyl sulfate polyacrylamide gel electrophoresis (SDS-PAGE) and analyzed by Western blot using the indicated antibodies. The SC5314 strain induced **(A)** a biphasic activation of host ERK 1/2 (the other isolates did not, not shown) and also **(B)** the phosphorylation of cortactin (pCort [Y466]) by SFKs. These are representative observations from at least three independent experiments. SC5314, 997,5 g, oral L3837 and blood L3881 isolates induced host cell signaling activation. HeLa cells were incubated with blastospores from four *C. albicans* isolates for the indicated time points. After incubation, HeLa cells were harvested, lysed, and SFK and cortactin activation were analyzed by Western blot using the indicated antibodies. Cortactin, SFKs and actin have different molecular weights (80–85, 60, and 45 kDa, respectively), thus the nitrocellulose membrane was cut into three horizontal strips, blocked and each strip was incubated with anti-pY466 cortactin, anti-pY416 SFK or anti-actin. **(C)** pSFK (Y416) and **(D)** pCort (Y466), cortactin phosphorylated by Src at the Y^466^ residue. Actin was used for protein loading normalization. Actin labeling is duplicated in **(C,D)**. pSFK/Act = densitometric rate of pSFK over actin. pCort/Act = densitometric rate of pCort over actin. These are representative observations from at least three independent experiments.

We first tested ERK 1/2 (one of the MAP kinase components) signaling in response to *C. albicans* SC5314 invasion in HeLa cells at several time points (Figure 3). As previously shown with TR146 oral epithelial cells by Moyes et al. (2010), *C. albicans* SC5314 also induced a biphasic MAP kinase response in another cell type, i.e., HeLa cells (Figure 3A) in a characteristic pattern: an increasing activation within the first 15 min (3–15 min), followed by a rapid decline (30 min), and increased activation again beginning at the 75 min time point, ending in a further decline of activation after 120 min (Figure 3A, 180 min). The initial activation was observed directly after contact with yeast cells, possibly due to the recognition of fungal cell wall components; the second activation phase corresponds to hyphae formation. However, such biphasic activation has not been observed with the other *Candida* isolates (not shown). We also tested cortactin phosphorylation at Y^466^ (phosphorylated site by SFKs) under the same conditions (Figure 3B). Cortactin phosphorylation at Y^466^ started to increase after 30–45 min and was sustained until 75 min of incubation, after which the signal declined.

### Src-family kinases (SFKs) and cortactin signaling during *C. albicans* invasion into HeLa cells

Once our experimental approaches using HeLa cells showed that *C. albicans* SC5314 induced a biphasic pattern of ERK signaling similar to the one obtained by Moyes et al. (2010) but in a different cell type, we evaluated SFK and cortactin signaling at selected time points in HeLa cells. Figures 3C,D shows that all *C. albicans* strains induced, at different levels, both SFK and cortactin phosphorylation in HeLa cells.

Regarding cortactin, 997,5 g, oral L3837 and blood L3881 strains also activated cortactin phosphorylation by Src at the Y^466^ residue; this phosphorylation was sustained for at least 180 min of *Candida*–HeLa cell contact. Lipid rafts have no impact in the epithelial cell invasion of all *Candida* isolates (Supplementary Figure 1A). Conversely, SFK inhibition negatively modulated all *Candida albicans* isolates invasion to HeLa cells (Supplementary Figure 1B).

## Discussion

*Candida albicans* is an opportunistic human pathogen that can cause superficial and hematogenous infections. The latter result in substantial morbidity and mortality and are a common, costly problem in healthcare settings. To access the systemic circulation, *C. albicans* has to invade both epithelial and endothelial barriers. For epithelial cells, two mechanisms have been described their invasion by *C. albicans*: active penetration and induced, actin-driven endocytosis. In this study, we have demonstrated that *C. albicans* strains—the reference strain SC5314 and three isolates with different clinical backgrounds—can invade HeLa cells and recruit cortactin and actin to sites of hyphae internalization, suggesting that at least part of the invasion process is mediated by induced endocytosis—which is dependent on dynamic microfilaments of the host—independently of the source of the clinical isolate. All four strains induced SFK/cortactin phosphorylation/activation, but to varying levels.

### Invasive ability, cortactin/actin recruitment, induction of Src/cortactin phosphorylation and survival under oxidative stress by *C. albicans* are independent of hyphal length or damage/internalization rates

In this study, all isolates of *C. albicans* from varying clinical backgrounds were able to invade and damage HeLa cells to different levels, and induced the accumulation of cortactin and actin around *C. albicans* hyphae that were in the process of being internalized by HeLa cells. Similar effects were observed for the reference strain, SC5314, in other epithelial cell types (Moreno-Ruiz et al., 2009). In addition, the invasive ability of *C. albicans* is apparently not related to the clinical background of the isolate.

It is noteworthy that 997,5 g strain—isolated from the blood of a patient who died within 24 h (unpublished results)—exhibited the greatest ability of all isolates tested to both internalize cells and cause damage *in vitro*.

Conversely, *C. albicans* L3881—isolated from the blood of a patient who had experienced candidemia but had undergone a clinical cure—expressed the *HWP1-2* allele and showed very reduced levels of *HWP1* mRNA expression (Padovan et al., 2009). In addition to displaying the shortest hyphae, as expected, *C. albicans* L3881 also showed the lowest rate of damage and internalization. Despite this, *C. albicans* L3881 was able to reach blood vessels, invade HeLa cells, recruit actin/cortactin, activate Src and cortactin by phosphorylation and, interestingly, survive under the pressure of nutritional and oxidative stress (i.e., ROS).

It is worth mentioning that many groups have previously shown the importance of hyphae length using *HWP1* mutants. For instance, Tsuchimori et al. (2000) have shown that a *HWP1* deletion mutant forms shorter hyphae and has decreased endothelial cell adherence, uptake and damage. Moreover, Wächtler et al. (2011) have examined a number of mutants for adhesion, internalization and damage of oral epithelial cells and have shown that a mutant with a HWP1 deletion has significant reduction in its epithelial cell adhesion and damage, but not internalization, which already indicates that hyphal length is likely to be important, but not an obstacle to cellular invasion. Indeed, in all cases, the deletions have been constructed artificially and the cell types used were different from this present study.

As a host defense mechanism, the production of ROS is a marked characteristic of phagocytic cells, which use such oxygen intermediates to kill invading pathogens. On one hand, *C. albicans* is relatively resistant to ROS, tolerating up to 20 mmol/L hydrogen peroxide (H_2_O_2_) under some conditions (Brown et al., 2014). Given that adaptation to oxidative stress is essential for pathogenicity, one can conclude that the inactivation of detoxifying enzymes, such as superoxide dismutases by *C. albicans* lead to severe attenuations in virulence and viability inside immune cells. Upon interaction with pathogens, phagocytes rapidly produce ROS. The ability to counteract oxidative attack by the patient would presumably aid fungal adaptation and invasiveness through the host. In the present study, L3881 isolate was resistant to oxidative stress and could reach the blood vessels of the patient, despite displaying a *HWP1* natural deletion and the shorter hyphae. It is an open question whether L3881 subverts the innate immune response by mediating the efficient detoxification of host-derived ROS, which ultimately allows it to reach deep tissues such as blood vessels.

Moreover, L3881 was also more resistant to EDTA than the SC5314 reference strain. This evidence indicates that the L3881 blood isolate has developed plasticity to deal with challenges and maintain its growth under different environmental conditions.

Cortactin possesses a series of C-terminal tyrosines that are heavily Src-phosphorylated and implicated in regulating actin remodeling during cell motility (Head et al., 2003). Phosphorylation of cortactin by Src greatly enhanced Arp2/3 complex-mediated actin polymerization (Tehrani et al., 2007). This event required Nck, an adaptor that links phosphorylated cortactin with neuronal WASp (N-WASp) and WASp-interacting protein (WIP) to activate the Arp2/3 complex, enhancing polymerization. Src phosphorylation of cortactin is important for actin polymerization during host cell invasion by some bacteria (*Shigella flexneri* and *Staphylococcus aureus*) or parasites (*Cryptosporidium parvum*) (Chen et al., 2003; Bougnères et al., 2004; Agerer et al., 2005).

The *C. albicans* reference strain and clinical isolates activated Src phosphorylation and consequently Src phosphorylation of cortactin (Y466) during HeLa cell invasion, thus pointing to a concerted series of events involving phosphorylation, and culminating in actin remodeling and actin-driven *C. albicans* invasion. Actin-driven invasion is a process that can be interrupted by both the up- and down-regulation of actin polymerization, thus drawing attention to the fact that actin polymerization must be carefully controlled to generate productive results; excessive assembly can bring the network to a halt (Haglund and Welch, 2011).

The tyrosine SFKs play a fundamental role in a wide variety of cellular processes including morphogenesis and proliferation, phagocytosis and host–pathogen interactions. In this study, it became clear that SFKs are important for *C. albicans* invasion, which has been evidenced by the decrease of internalization rate of all *Candida* isolates into HeLa cells when using a SFK inhibitor. SFKs normally drive actin-dependent rearrangements required for cellular migration and may be used by the fungus to promote cell invasion in the same way of several bacteria (Mounier et al., 2009). Upon pathogen-cell adhesion, SFKs may associate to and activate other molecules such as FAK to regulate several actin-related processes, including phosphorylation of key substrates such as paxillin and cortactin (Ferreira et al., 2016). It has also been shown that activation of host cell SFK is correlated to the actin dependent internalization of bacterial pathogens (Pizarro-Cerdá et al., 2016). Src-dependent remodeling of the actin cytoskeleton has been also reported for viruses and parasites, suggesting that such signaling corresponds to a common feature that controls actin dynamics at the membrane (Mitra and Schlaepfer, 2006; Munter et al., 2006). As far as we know this is the first time that the inhibition of Src-familiy kinases has been shown to hinder host cell invasion by *C. albicans*, pointing to a key role for SFK in driving actin polymerization and consequently the internalization of *C. albicans*.

Several pathogens have been described to enter professional phagocyte cells via cholesterol-enriched membrane lipid raft microdomains. In fact, *C. albicans* uptake by human monocyte has been strongly impaired by the cholesterol extracting agent methyl-β-cyclodextrin in the study by de Turris et al. (2015). It is worth mentioning that HeLa cells are not professional phagocytes as monocytes or macrophages. This is probably why the disruption of lipid rafts in HeLa cells did not impact on *C. albicans* internalization.

Collectively, these results suggest that *C. albicans* invasion of HeLa epithelial cells—and also the ability to reach and adapt to live in blood—may be independent of fungal adherence and hyphal length, or even the ability to damage cells, since L3881, isolated from a patient's blood, has been able to both invade cells and, recruit actin/cortactin, activate SFKs and cortactin, survive under nutritional and oxidative stress and reach blood tissues, despite its shorter hyphae.

### Final consideration—building a niche

When discussing *C. albicans* invasion, it is known that to infect mucosal tissues, gain access to blood and escape blood flow, *C. albicans* must interact with both epithelial and endothelial cells, respectively (Davis, 2009). In superficial infections (skin and mucosa), this fungus only reaches blood vessels when a mechanical disruption (wound or cut) to epithelial tissue occurs.

Interestingly, oropharyngeal candidiasis is prevalent in HIV-positive patients and, although rare (especially in the terminally ill), HIV-positive patients can develop systemic candidiasis. Regarding the mechanisms involved in *Candida* penetration of epithelial cells, what is known from *in vitro* experiments is that active penetration is the predominant form of invasion, while induced endocytosis seems to occur in the initial phases of tissue colonization (Wächtler et al., 2012).

The fungus uses a variety of strategies in order to survive; other types of interactions may exist *in vivo* that we have not yet seen *in vitro*. In systemic infections, it is believed that the gastrointestinal tract is a major source of *Candida* invasion in addition to catheters or surgery, for example (Perlroth et al., 2007). The basic pH of gastrointestinal tissue, its 37°C temperature, ample contact with epithelial cells and an iron-restricted environment are strong inducers of hyphae formation, i.e., this fungus always forms hyphae when it meets its host (Almeida et al., 2008). However, in infected organs, hyphae, yeasts and pseudohyphae can be observed by histological analysis, probably because yeasts are part of the proliferation process (the more yeasts present, the more hyphae formed). However, the importance of pseudohyphae remains unknown.

Hematogenous infections caused by pathogens of the *Candida* genus have increased heavily in Latin America (Doi et al., 2016) mainly because modern medical treatments are becoming more invasive and more people are being treated with immunosuppressive drugs. Hematogenous candidiasis is a silent disease. Often, clinicians are only able to detect the infectious agent when the patient already has an irreversible condition. The mortality rate for hematogenous candidiasis is 40–60%, which are devastating numbers. However, drugs commonly used to control the infection cause pathogen resistance. Epithelial cells play a key role in many human–microbiota and/or pathogens interactions. Interactions of these cells with *C. albicans* have been shown to be critical to host and fungal cell responses (Moyes et al., 2015). Molecules of both *Candida* and the host are important for *Candida* internalization into epithelial cells, which is the first step to further dissemination leading to hematogenic infections. A better elucidation of these factors may significantly contribute to the control of systemic infections in hospitals, such as the rational development of new compounds against the essential components triggered during the invasion of host cells by the fungus.

## Author contributions

Conception of the study: DB. Designed the experiments: PM, AB, AP, FS, RA, ES, and DB. Performed the experiments: PM, AB, CO, LR, TM. Interpretation of the results and data analysis: PM, AB, AP, RM, FS, RA, ES, and DB. Contributed reagents/materials/analysis tools: AP, RM, FS, RA, ES, and DB. Wrote the manuscript: PM, AB, and DB. All authors read and approved the final manuscript.

### Conflict of interest statement

The authors declare that the research was conducted in the absence of any commercial or financial relationships that could be construed as a potential conflict of interest.
