# Supplementary material for: Candida albicans: The Ability to Invade Epithelial Cells and Survive under Oxidative Stress Is Unlinked to Hyphal Length
Source: Front Microbiol. 2017 Jul 17;8:1235. doi: 10.3389/fmicb.2017.01235 (PMC5511855; doi:10.3389/fmicb.2017.01235)
Supplement: Supplementary file 1 [file DataSheet1.pdf]

Supplementary Material

Supplementary Table 1: Characteristics of *in vitro* HeLa cell invasion of *C. albicans* isolates used in this study.

**Number**	**Isolation**	**Hyphal length**	**Invasion into HeLa cells**	**Actin/cortactin recruitment; SFK/cortactin phosphorylation**	**References**
SC5314	Blood, wild type, patient with disseminated candidiasis. Reference strain.	Normal	High invasion and cytotoxicity rates	Yes	(Gillum et al., 1984); (Moreno-Ruiz et al., 2009), this study
997,5 g	Blood, patient had candidemia, died.	Normal	Very high invasion and cytotoxicity rates	Yes	(Chaves et al., 2012), this study
L3881 (previously named L757)	Blood, patient had candidemia, evolved to clinical cure. Natural *HWP1* mutant.	Very short	Low invasion rate, no cell cytotoxicity	Yes	(Chaves et al., 2012), (Padovan et al., 2009), this study
L3837	Oropharyngeal	Normal	Low invasion rate, low cell cytotoxicity	Yes	This study

A

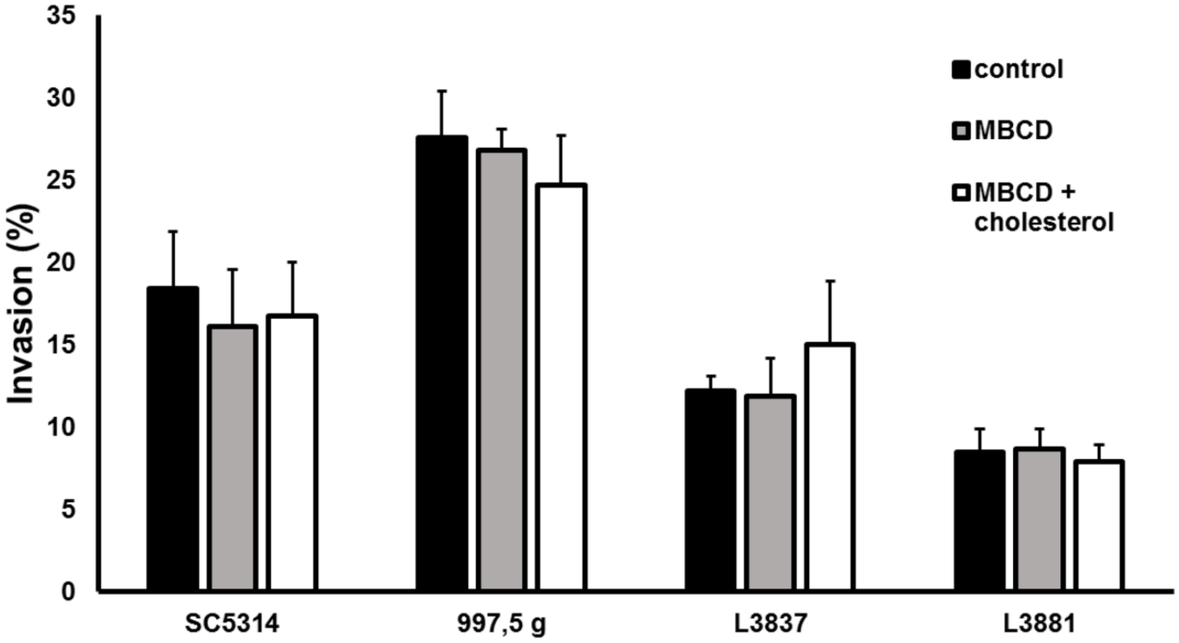

B

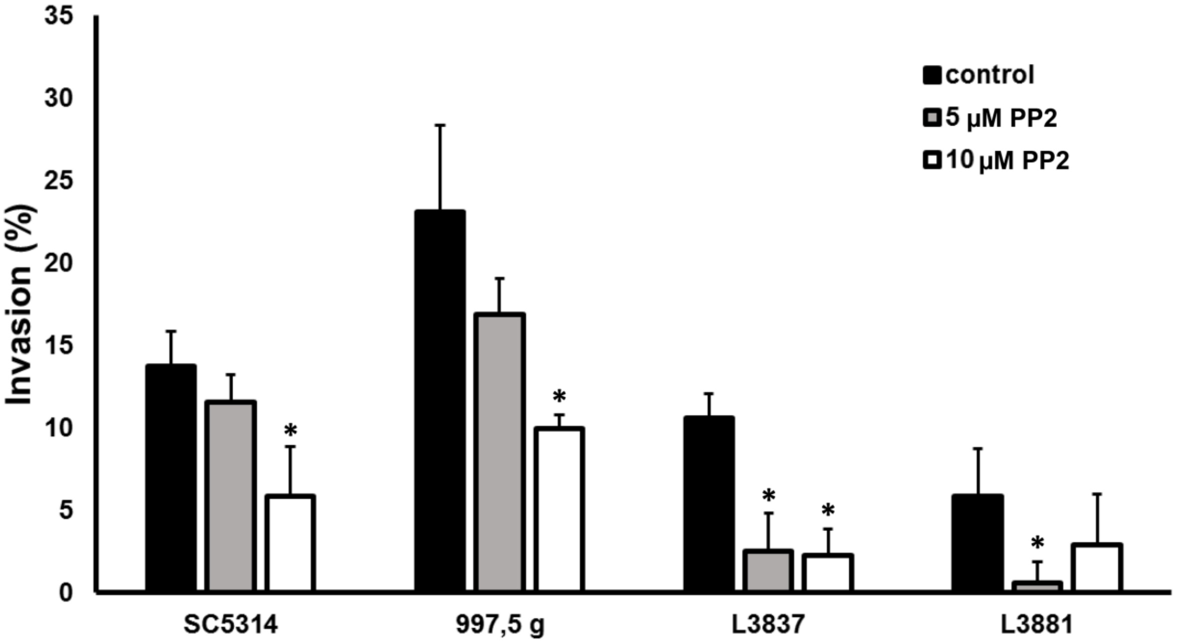

Supplementary Figure 1: Cell invasion assay in presence of methyl-β-cyclodextrin MβCD (A) to deplete the cholesterol and Src-Family Kinase (SFK) inhibitor, PP2 (B). To evaluate whether epithelial cell lipid rafts are involved on cell invasion by *C.*

albicans, HeLa cells were incubated with 10 mM MβCD (Sigma-Aldrich) for 1 h or with 10 mM MβCD for 1 h and 1 mM cholesterol-MβCD complex (water-soluble cholesterol – Sigma Aldrich) for 30 min. Next, epithelial cells were washed three times with RPMI 1640 without FBS. To determine the importance of SFK activation on cell invasion, HeLa cells were incubated for 2 h in RPMI 1640 without FBS containing 5 or 10 μM PP2 (Calbiochem) or with 0.05% DMSO (vehicle for PP2). Then, 2×10^5 *C. albicans* blastospores were added to HeLa cells and incubated for 2 h at 37 ^{o}C in 5% CO_2. After incubation with *C. albicans*, cell invasion was evaluated as described in Materials and Methods.
